# Identification of key genes and the pathophysiology associated with allergen-specific immunotherapy for allergic rhinitis

**DOI:** 10.1186/s12865-023-00556-1

**Published:** 2023-07-10

**Authors:** Kai Fan, Shican Zhou, Ling Jin, Shiwang Tan, Ju Lai, Zimu Zhang, Jingwen Li, Xiayue Xu, Chunyan Yao, Zhiqiang Yan, Shaoqing Yu

**Affiliations:** 1 Department of Otorhinolaryngology-Head and Neck Surgery, Tongji Hospital, School of Medicine, Tongji University, Shanghai, 200065 China; 2 Department of Allergy, Tongji Hospital, School of Medicine, Tongji University, Shanghai, 200065 China; 3 Department of Otolaryngology Head & Neck Surgery, The Affilicated Huaihai Hospital of Xuzhou Medical University, 236 Tongshan Road, Xuzhou, 221004 Jiangsu China

**Keywords:** Allergen-specific immunotherapy, Allergic rhinitis, Bioinformatics analysis, Differentially expressed genes, microRNAs.

## Abstract

**Background:**

Allergen-specific immunotherapy (AIT) is a causative treatment in allergic rhinitis (AR), comprising long-term allergen administration and over three years of treatment. This study is carried out for revealing the mechanisms and key genes of AIT in AR.

**Methods:**

The present study utilized online Gene Expression Omnibus (GEO) microarray expression profiling dataset GSE37157 and GSE29521 to analyze the hub genes changes related to AIT in AR. Based on limma package, differential expression analysis for the two groups (samples of allergic patients prior to AIT and samples of allergic patients undergoing AIT) was performed to obtain differentially expressed genes (DEGs). Gene Ontology (GO) analysis and Kyoto Encyclopedia of Genes and Genomes (KEGG) pathway analysis of DEGs were conducted using DAVID database. A Protein-Protein Interaction network (PPI) was built and a significant network module was acquired by using Cytoscape software (Cytoscape, 3.7.2). Utilizing the miRWalk database, we identified potential gene biomarkers, constructed interaction networks of target genes and microRNAs (miRNAs) using Cytoscape software, and explore the cell type-specific expression patterns of these genes in peripheral blood using publicly available single-cell RNA sequencing data (GSE200107). Finally, we are using PCR to detect changes in the hub genes that are screened using the above method in peripheral blood before and after AIT treatment.

**Results:**

GSE37157 and GSE29521 included 28 and 13 samples, respectively. A total of 119 significantly co-upregulated DEGs and 33 co-downregulated DEGs were obtained from two datasets. The GO and KEGG analyses demonstrated that protein transport, positive regulation of apoptotic process, Natural killer cell mediated cytotoxicity, T cell receptor signaling pathway, TNF signaling pathway, B cell receptor signaling pathway and Apoptosis may be potential candidate therapeutic targets for AIT of AR. From the PPI network, 20 hub genes were obtained. Among them, the PPI sub-networks of CASP3, FOXO3, PIK3R1, PIK3R3, ATF4, and POLD3 screened out from our study have been identified as reliable predictors of AIT in AR, especially the PIK3R1.

**Conclusion:**

Our analysis has identified novel gene signatures, thereby contributing to a more comprehensive understanding of the molecular mechanisms underlying AIT in the treatment of AR.

## Introduction

Allergic Rhinitis (AR) is a common inflammatory disease of the nasal mucosa, affecting 20–30% of the populations of different countries [1–3]. During the past three decades, the rapid industrialization and related changes in environment and lifestyle might have led to an increase in the incidence rate of AR worldwide [3]. The current recommended treatment for AR mainly includes allergen avoidance, pharmacotherapy, Allergen-specific immunotherapy (AIT), and patient education [4]. Although many drugs are effective and without significant side effects, drugs represent a symptomatic treatment, while AIT represents the only curative and specific treatment approach and might alter the natural course of the disease [5, 6].AIT is an etiology-based treatment, aimed at inducing tolerance to allergens, such as pollen, dust mites or moulds, by administering increasing amounts of the causative allergen through subcutaneous or sublingual route [7]. The AIT has been used as a desensitization and potential cure therapy for AR for nearly a century [8], which can be administered subcutaneously (SCIT) or sublingually (SLIT). Both routes of administration are safe, effective, and can lead to tolerance lasting years after treatment cessation. Both routes of administration are effective and safe, and the tolerability can be guaranteed for several years after the cessation of treatment.

The evidence of efficacy and safety of AIT is high, the issue of cost and time commitment must be taken into account. High levels of compliance and persistence of patients are crucial to achieving the desired clinical effects. Retrospective studies provide some evidence that AIT typically involves over a 3 to 5 years treatment course, as AR is a chronic condition [7, 9, 10]. The cumulative costs of AIT in AR can be significant over time. There is a great need to explore the immunological mechanisms of action of AIT to shorten the burden of AR treatment. The mechanisms by which AIT mediates its anti-inflammatory effects remain incompletely defined [11]. Data from previous studies have shown that AIT modifies the responses of antigen-presenting cells, T cells, and B cells as well as both the number and the function of effector cells that mediate the allergic response [6, 12], such as a shifting of the cytokine milieu from Th2 to Th1 predominance, decreased recruitment and activation of eosinophils at sites of allergen challenge following AIT [9, 13]. The induction of an immune tolerance state represents an essential step in AIT [14], how the mechanism responsible for AIT improves clinical symptoms of AR is still not entirely clear. In order to provide an important contribution to the evidence regarding AIT in AR an reduce the burden of patients, we compare and analyze the original genetic data of the patients prior to AIT and patients undergoing AIT, hope to excavate potential key genes and microRNAs(miRNAs), and reveal the treatment pathogenesis at the molecular level of AIT in AR.

## Results

### Identification of differentially expressed genes

**Fig. 1:**
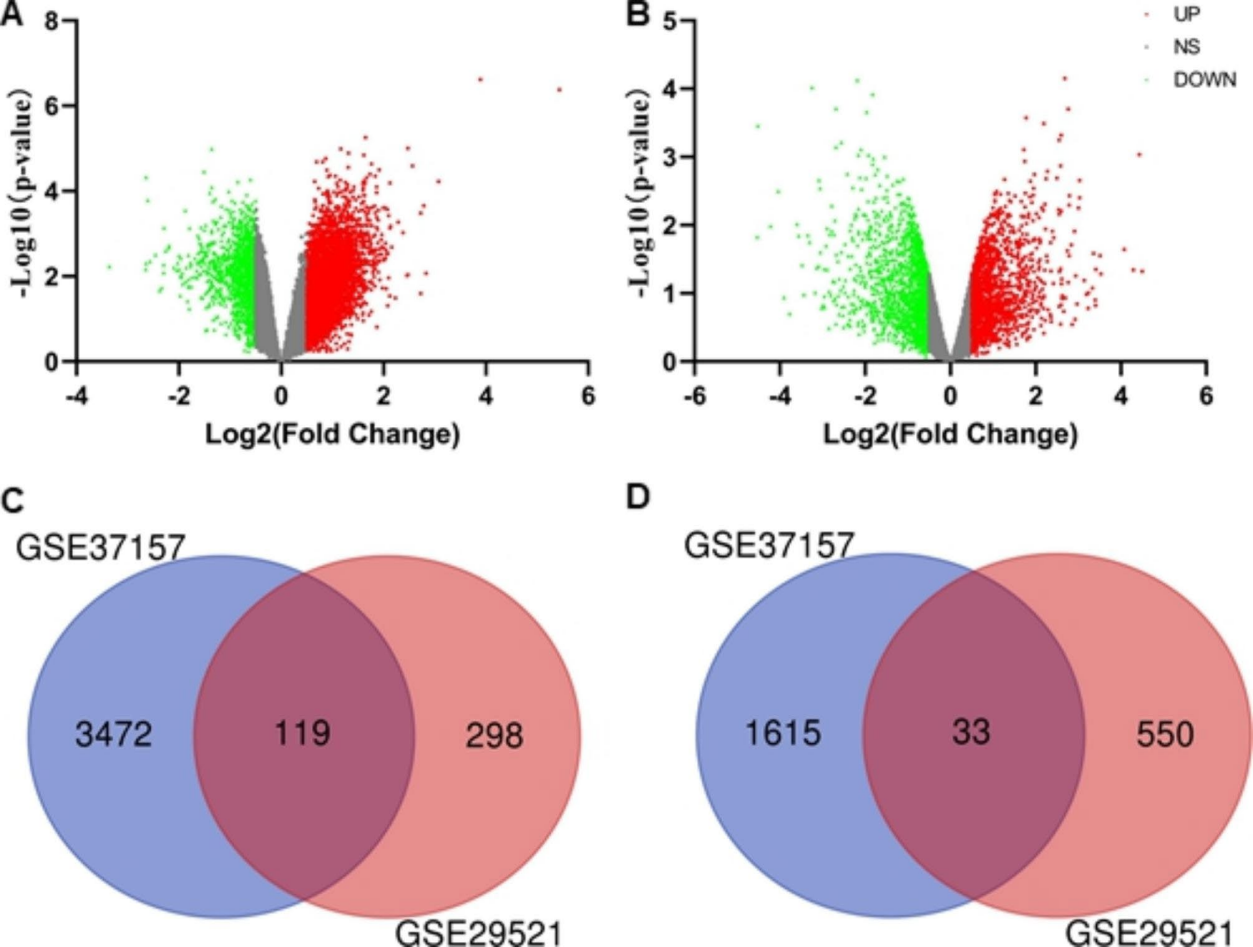
Differentially Expressed Genes Before and after AIT **A-B** The volcano plot for the differentially expressed genes (DEGs). The horizontal axis and vertical axis separately represent log2 fold change and -log10 P value. Red points represented up-regulated genes, while green points represented down-regulated genes. Black points represented genes with no significant difference (NS). **A** GSE37157 and **B** GSE29521 datasets. **C-D** Venn diagrams illustrating the number of **C** upregulated and **D** downregulated genes in the two datasets. The intersection in violet represents the DEGs that are common between the two datasets

Datasets GSE37157 and GSE29521 from GEO were selected for this study based on two critical criteria: the presence of individuals in the maintenance phase of AIT and the exclusive inclusion of PBMC samples from AR patients. The volcano plots, shown in Fig. 1A and B, were drawn based on the analysis of gene expression in each data set. After removing duplicate genes and expression values lacking specific gene symbols, we obtained 119 jointly up-regulated DEGs and 33 jointly down-regulated DEGs from the two datasets (*P* value < 0.05 and |logFC|> 0.5) (Fig. 1C, D). The number of co-expressed up-regulated DEGs is greater than the number of co-expressed down-regulated DEGs.

### GO and KEGG analysis of DEGs

We uploaded the 152 co-DEGs (119 jointly up-regulated DEGs and 33 jointly down-regulated DEGs) to perform the GO analysis and KEGG pathway analysis. The outcomes showed that protein transport (GO:0015031; P-value = 4.78E-04) was the most significantly enriched, followed by positive regulation of apoptotic process (GO:0043065; P-value = 9.34E-04) (Table 1). Meanwhile, KEGG pathway enrichment analysis revealed that Hepatitis B (hsa05161; P-value = 4.01E-04), Natural killer cell mediated cytotoxicity (hsa004650; P-value = 9.67E-04), T cell receptor signaling pathway (hsa04660; P-value = 0.014464904), TNF signaling pathway (hsa04919; P-value = 0.000616), Apoptosis (hsa04210; P-value = 0.020497316) and B cell receptor signaling pathway (hsa04662; P-value = 0.018129562) were significantly enriched, as shown in Table 2.

**Table 1:** The top five GO terms in enrichment analyses of DEGs.

Category	Pathway ID	Pathway description	Count	P-Value
Biological Process	GO:0015031	protein transport	12	4.78E-04
Biological Process	GO:0043065	positive regulation of apoptotic process	10	9.34E-04
Biological Process	GO:0000722	telomere maintenance via recombination	4	0.002356991
Biological Process	GO:0019985	translesion synthesis	4	0.003311553
Biological Process	GO:0006919	activation of cysteine-type endopeptidase activity involved in apoptotic process	5	0.005113391
Cellular Component	GO:0005654	nucleoplasm	45	2.14E-06
Cellular Component	GO:0016020	membrane	37	1.17E-05
Cellular Component	GO:0005829	cytosol	48	1.70E-05
Cellular Component	GO:0005737	cytoplasm	61	4.34E-04
Cellular Component	GO:0016607	nuclear speck	7	0.005119236
Molecular Function	GO:0005515	protein binding	98	2.33E-05
Molecular Function	GO:0004222	metalloendopeptidase activity	6	0.00250407
Molecular Function	GO:0008134	transcription factor binding	9	0.002563392
Molecular Function	GO:0001047	core promoter binding	4	0.016095866
Molecular Function	GO:0019899	enzyme binding	8	0.021136619

**Table 2:** The top 20 pathways in terms of KEGG analyses of DEGs.

Category	Pathway ID	Pathway description	Count	P-Value
KEGG PATHWAY	hsa05161	Hepatitis B	8	4.01E-04
KEGG PATHWAY	hsa05166	HTLV-I infection	10	5.79E-04
KEGG PATHWAY	hsa04650	Natural killer cell mediated cytotoxicity	7	9.67E-04
KEGG PATHWAY	hsa03030	DNA replication	4	0.004602972
KEGG PATHWAY	hsa05133	Pertussis	5	0.005341509
KEGG PATHWAY	hsa04010	MAPK signaling pathway	8	0.009455073
KEGG PATHWAY	hsa04932	Non-alcoholic fatty liver disease (NAFLD)	6	0.013692686
KEGG PATHWAY	hsa04660	T cell receptor signaling pathway	5	0.014464904
KEGG PATHWAY	hsa04668	TNF signaling pathway	5	0.018129562
KEGG PATHWAY	hsa03430	Mismatch repair	3	0.019576268
KEGG PATHWAY	hsa04725	Cholinergic synapse	5	0.020461572
KEGG PATHWAY	hsa04210	Apoptosis	4	0.020497316
KEGG PATHWAY	hsa04670	Leukocyte transendothelial migration	5	0.02297114
KEGG PATHWAY	hsa04722	Neurotrophin signaling pathway	5	0.026362394
KEGG PATHWAY	hsa04662	B cell receptor signaling pathway	4	0.027108064
KEGG PATHWAY	hsa05169	Epstein-Barr virus infection	5	0.027799041
KEGG PATHWAY	hsa04917	Prolactin signaling pathway	4	0.029182478
KEGG PATHWAY	hsa04380	Osteoclast differentiation	5	0.034838908
KEGG PATHWAY	hsa05203	Viral carcinogenesis	6	0.043708607
KEGG PATHWAY	hsa04015	Rap1 signaling pathway	6	0.047640557

### PPI network analysis and identification of hub genes

**Fig. 2:**
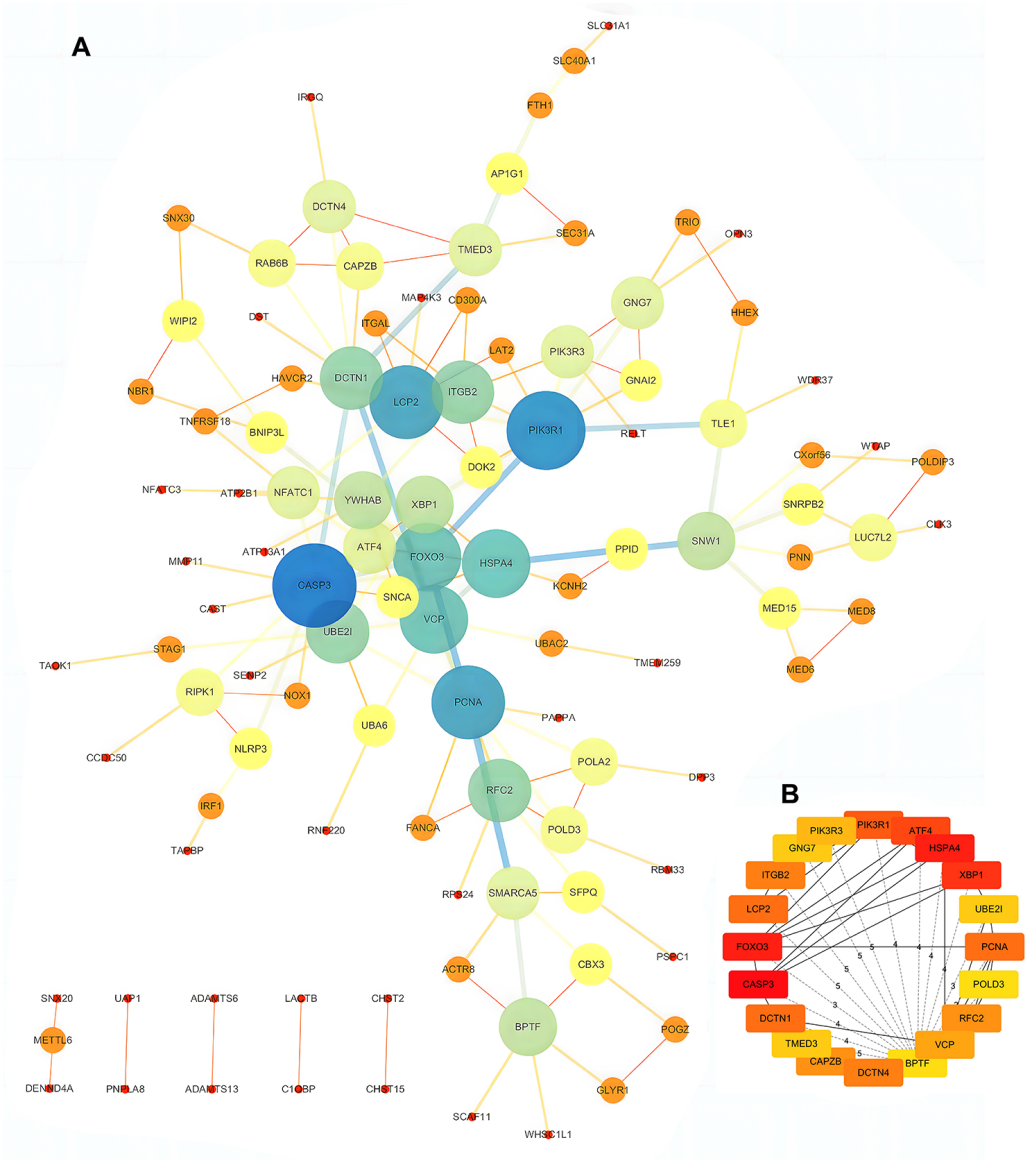
PPI network analysis and hub gene identification were conducted. **A** PPI network of DEGs. **B** CytoHubba plugin was used to analyze hub genes with MCC.

To isolate core genes from the co-DEGs, PPI network analysis was conducted on the complete set of DEGs using STRING. Visualization of results was facilitated using Cytoscape software.In the PPI analysis, the connections between nodes represent the interactions between the proteins encoded by co-DEGs, which included 152 nodes and 154 edges (Fig. 2A). The cytoHubba plugin (http://apps.cytoscape.org/apps/cytohubba) was then used to analyze hub genes with MCC (Maximal Clique Centrality), and genes with the top 20 scores were identified as hub genes. Among these genes, CASP3, HSPA4, FOXO3, XBP1, ATF4, PIK3R1, DCTN1, LCP2, PCNA, DCTN4, ITGB2, CAPZB, RFC2, VCP, PIK3R3, UBE2I, TMED3, GNG7, POLD3, and BPTF showed the highest node scores and were identified as hub genes (Fig. 2B).

### miRNA interaction network analysis

**Fig. 3:**
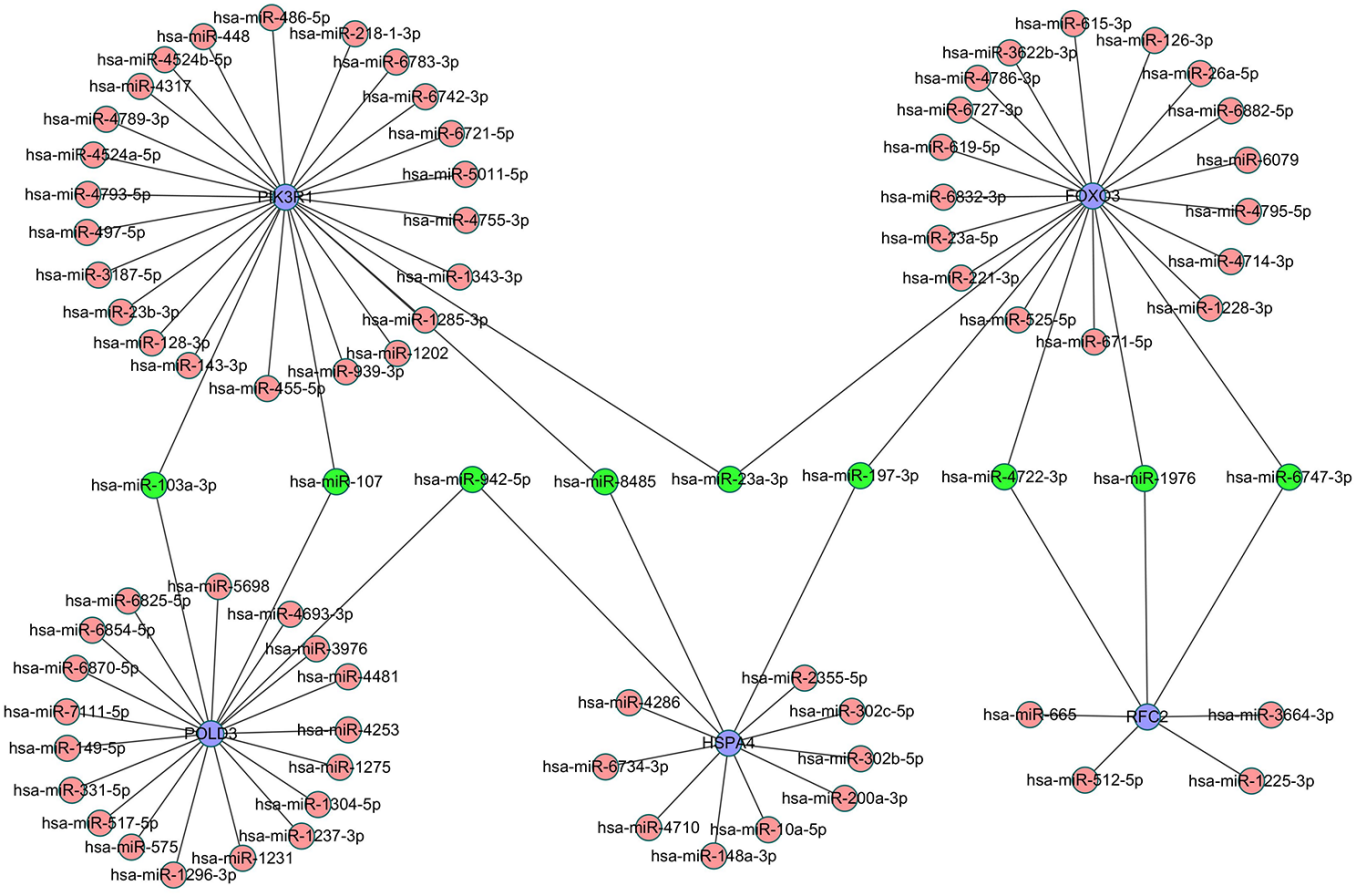
Interaction network between genes and their targeted miRNAs. Genes are colored in blue; miRNAs are colored in red; miRNAs targeting more than two genes are colored in green

After selecting 20 genes related to AIT cytokine signal of AR, miRNA analysis was performed on selected genes through miRWalk v3.0 software (http://zmf.umm.uni-heidelberg.de/apps/zmf/mirwalk/).The results turned out that 89 gene-miRNA pairs were contained in the interaction network and these were visualized by Cytoscape software. Through analysis of the hub genes, several potential targets were identified along with their associated miRNAs: PIK3R1 with 27, FOXO3 and POLD3 with 22 and 21, and HSPA4 and RFC2 with 12 and 7 miRNAs each. (Fig. 3).

### Hub genes expression at the single cell level

**Fig. 4:**
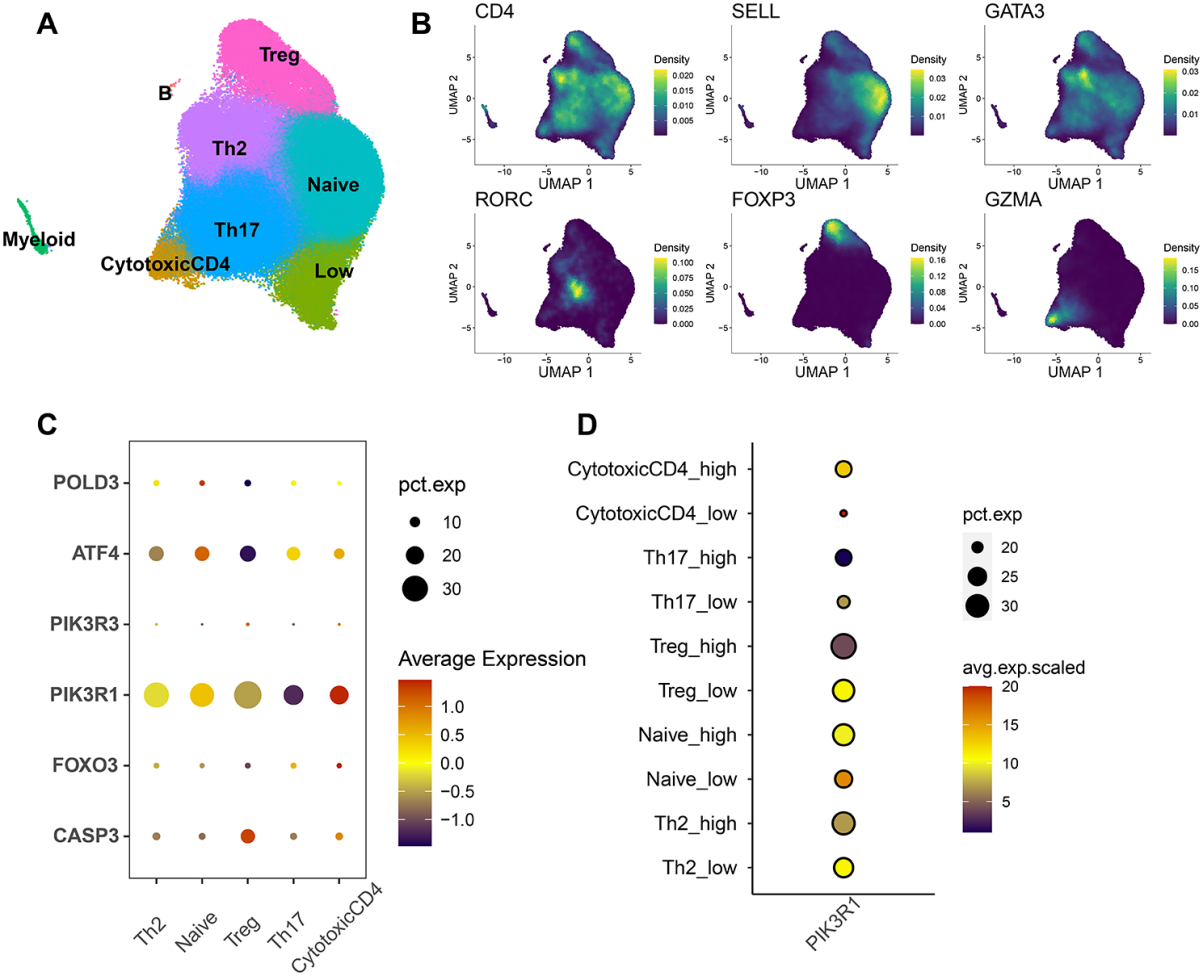
Hub Gene expression at the single cell level. **A-B** The expression levels of known transcription factors and marker genes were used to identify five major T-cell clusters (Naive, Treg, Th2, Th17, and cytotoxic CD4 cells), which are depicted in UMAP. **C** Expression of hub gene in different cell types. **D** the expression of PIK3R1 in different curative effects

To examine the cell type-specific expression patterns of the hub genes in peripheral blood, we analyzed publicly available single-cell RNA sequencing data (GSE200107). On the basis of the expression levels of known transcription factors and marker genes, 5 main T-cell clusters (Naive, Treg, Th2, Th17, and cytotoxic CD4 cells) were identified and are shown in UMAP (Uniform Manifold Approximation and Projection) (Fig. 4A, B). The Dotplot showed that PIK3R1 had higher expression in different T-cells and the expression of PIK3R1 in the high curative effect group was higher than the low (Fig. 4C, D).

### Peripheral blood hub gene mRNA expression levels in patients before and after immunotherapy

**Fig. 5:**
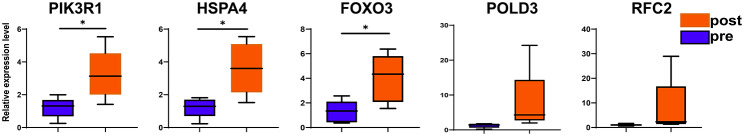
Hub gene mRNA expression levels in patients before and after immunotherapy. *P < 0.05

As showcased in Fig. 5, Peripheral blood FOXO3, PIK3R1 and HSPA4 expression were significantly higher in patients with allergic rhinitis after 6 months of immunotherapy, while RFC2 and POLD3 expression were not significantly different.

## Discussion

AR is one of the most popular chronic respiratory diseases in the world. Global Burden of Disease study has determined that it has affected the health of millions of people and reduced the quality of life of patients in the past century [15, 16]. SCIT and SLIT immunotherapy are the two most widely prescribed AIT routes worldwide [17]. Intralymphatic and epicutaneous routes, although less common, offer potential benefits including reduced treatment duration and improved patient comfort [18]. Despite the field of AIT is experiencing exciting and novel developments for the treatment of AR, there are several limitations. On the one hand, AIT has a significant economic burden on patients and need high levels of compliance and persistence; On the other hand, we still lack an understanding of the distinctive immunological mechanisms compared with many other diseases. Genetic insights have transformed stagnation into advance with a real scientific basis and made mechanisms more tractable and understandable. The rapidly developing and extensively using of microarray technology has revealed thousands of genes during immunotherapy in AR, which may provide promising targets for AR treatment [19]. Thus, it is necessary to identify biomarkers and provide proof of principle for AIT in AR. The present study employed bioinformatics approaches to elucidate the essential genes and pathways associated with AIT in AR.To achieve this, GSE37157 and GSE29521 microarray datasets were included to analyze DEGs and identified hub genes. In total, 119 jointly up-regulated DEGs and 33 jointly down-regulated DEGs were identified from two datasets. The Go analysis results of AIT for AR showed that the module changes mainly focused on the protein transport, positive regulation of apoptotic process. The KEGG enrichment analysis of DEGs showed that AIT of AR involves metabolic pathways in natural killer cell mediated cytotoxicity, T cell receptor signaling pathway, TNF signaling pathway, B cell receptor signaling pathway, and apoptosis. In addition, the 20 high scoring genes in the PPI network were defined as hub genes. For the sake of enhancing our understanding of the molecular mechanisms, integrated network analysis of gene-miRNA interactions was performed. Our results provide new research hypotheses that these enriched modules and pathways are involved in the AIT of AR. These findings could help researchers to improve the understanding of AR and treatment mechanisms, and also provide new ideas for further research.

It is well-known that AR is an IgE-mediated hypersensitivity disease caused by inhalation that is able to promote IgE synthesis and mast cell proliferation [20]. The previous study has shown that activation of FcepsilonRI receptors in mast cells can promote the release and synthesis of inflammatory mediators, and provide proliferation and survival probability, all of the above conditions are related to protein transport [21]. The point of view is in conformance with our results that DEGs were associated with protein transport in the GO analysis. Apoptotic cell death is an important mechanism for maintaining homeostasis in the immune system, and for regulating the fates of lymphocytes following encounters with self and foreign antigens [22]. According to GO analysis results, we speculate that the positive regulation of apoptotic process might be involved in the progression of AIT in AR.

In the KEGG pathway analysis, DEGs were enriched for B cell receptor signaling, T cell receptor signaling, and TNF signaling pathways. To date, the key factors to promoting an adaptive immune response including induction of allergen-specific blocking antibodies, regulatory T and B cells, and immunosuppressive cytokines [23, 24], these studies are consistent with metabolic pathway findings. Recent data demonstrated that miR-375 can prevent nasal mucosal cell apoptosis by inhibiting JAK2/STAT3 pathway, thereby improving the symptoms of AR [25]. Further study suggested that the blockade of PD-1/PD-L1 pathway promotes the apoptosis of CD19 + CD25 + Bregs and suppresses the secretion of IL-10 in patients with AR [26]. Wang H and colleagues reported in their 2010 study that allergen exposure to peripheral blood mononuclear cells, derived from patients suffering from seasonal Allergic Rhinitis (AR), augmented the expression of IL-17RB, a key modulator of basophil apoptosis and degranulation[27]. The above results suggest that the apoptosis of inflammatory cells (Bregs, basophil) plays a key role in the immunotherapy of AR.

In the PPI network analysis, 20 DEGs were identified as hub genes, in addition to PIK3R3, GNG7, and POLD3, all other hub genes were upregulated. In recent years, many studies have found that Caspase-3 (CASP3) plays an important role in apoptosis in the field of cancer research [28]. The results are consistent with our microarray analysis, in which CASP3 were identified as DEGs in the progression of TNF signaling pathway. It is well known that members of the tumor necrosis factor (TNF) receptor superfamily contribute to immune up-regulation by a mechanism of action interactions [29]. Accumulating evidence in recent years indicates that the TNF and TNF receptor (TNFR) promote differentiation, clonal expansion, and survival of antigen-primed CD4 + and CD8 + T cells, and have a pivotal role in T-cell-mediated adaptive immunity and diseases [30]. Although there have been few studies on the CASP3 and immunotherapy for AR, we speculate that CASP3 might take part in the progression of TNF signaling pathway of cellular immunity. The subnetworks of ATF4, PIK3R1, and PIK3R3 filtered out from our study have been also shown to participate in TNF signaling pathway, since these hub genes involve immune regulation in cancer and AR disease [31].

The miRNAs are endogenous non-coding RNA molecules with a length of 18–22 nt, target the 3’UTR region of genes, which can regulate gene expression, degrade target genes, and inhibit post-transcriptional translation [32]. In recent years, circRNAs, as a new molecular marker, have been attracting more and more attention for their important role in the regulation of cellular information [29]. Utilizing gene-miRNA results from the predictive analysis tool, the target pairs of human gene-miRNA for AIT of AR were identified, revealing PIK3R1 as a likely target of 27 differentially expressed miRNAs. Single-cell RNA sequence analysis also revealed the unique status of PIK3R1. Research has shown that PIK3R1 is a potential target to evaluate cancer-specific molecular pathways and their correlation with tumor immune profile in cancer [33]. In view of tumors and allergic rhinitis both involve complex immune regulatory systems, these results indicate that PIK3R1 may be an important target of AIT of AR. Interestingly, another research shows that the FOXO3 is a gene involved in the etiology of a number of respiratory diseases, and it is proved to be associated with asthma and allergic rhinitis [34], which is consistent with our research results that FOXO3 is an important hub gene of AIT for AR. In addition, hsa-miR-103a-3p and hsa-miR-107 were found to enrich the cancer induction pathway and participate in the regulation of innate immune response [24, 35]. It is speculated that the above 9 miRNAs might be related to the differences of AIT for AR, the specific molecular mechanism remains to be further studied.

## Conclusion

This study used gene expression data sets (GSE37157 and GSE29521) to determine the differential expression of genes related to the progression of AIT in AR, providing a reliable comprehensive analysis for the study of disease and treatment mechanisms. A total of 119 co-upregulated DEGs, 33 co-downregulated DEGs, and 20 hub genes were obtained, and these findings may become new potential targets of AIT in AR. The GO and KEGG analyses demonstrated that protein transport, positive regulation of apoptotic process, Natural killer cell mediated cytotoxicity, T cell receptor signaling pathway, TNF signaling pathway, B cell receptor signaling pathway, and Apoptosis may be potential targets for immunotherapy of AR. Deeper insights into the full range of molecules have been achieved through this study, leading to the identification of several key genes, including CASP3, FOXO3, PIK3R1, PIK3R3, ATF4, and POLD3. These results may help to provide new targets and novel therapeutic strategies for immunotherapy in AR. However, the underlying molecular mechanism of immunotherapy for allergic rhinitis has not been fully elucidated. Future investigations will require further experimental validation to corroborate alterations in gene expression. Collection of biological samples and behavioral data from patients before and during AIT is of paramount importance for enhancing the depth of ensuing research.

## Materials and methods

### Public datasets

In order to obtain gene expression data sets, we use the Gene Expression Omnibus (GEO) database, which is a public online genomics data containing high-throughput gene expression data, chips, and microarrays [36]. To identify the candidate genes of AIT in AR, we selected the AR patients of the GSE37157[37] and GSE29521[38] datasets for further study. The microarray dataset GSE37157 was deposited by Cárdaba B et al., and a total of 12 samples were utilized, including 6 samples of PBMC from patients undergoing AIT, 6 samples of patients prior to AIT. The microarray dataset GSE29521 was deposited by Davis L, and 6 samples were included in the analysis, containing 3 samples of PBMC from patients undergoing AIT, 3 samples of PBMC from patients prior to AIT. The probes were converted into the corresponding gene symbol according to the annotation information in the platform. All of the data were freely available online. In addition, we selected the AR patients of the GSE200107[39] datasets for further study. The datasets included 14 PBMC samples collected from 7 patients suffering from Japanese cedar pollinosis before and 1 year after SLIT treatment.

### Data processing and identification of DEGs

In this study, we performed the online analytical tool GEO2R for differential expression analysis. The significant DEGs were defined as the absolute log2 value of the fold change of gene expression > 0.5, and the P value < 0.05. GSE37157 samples and GSE29521 samples were analyzed independently in the data processing and identification, and the DEGs were determined by the intersection of the two datasets. We use the online tool Venn diagram to draw the Venn diagram of DEGs (http://bioinformatics.psb.ugent.be/webtools/Venn).

### Functional and pathway enrichment analysis

We used DAVID (http://DAVID.org) [40] to perform the functional enrichment analysis of DEGs. DAVID is regarded as the most common functional enrichment analysis of DEGs: for Gene Ontology (GO) functional enrichment analysis and Kyoto Encyclopedia of Genes and Genomes (KEGG) pathway enrichment analysis. GO analysis was used to predict protein function, including three modules, namely, biological process (BP), molecular function (MF), and cell composition (CC) [41]. By using KEGG pathway analysis, the deg will be integrated into a specific pathway to construct networks of molecular interactions, molecular reactions and mutual relationships [42]. We uploaded the significant genes to investigate the potential functions with the threshold of P < 0.05.

### Protein-protein Interaction (PPI) network

The PPI network analysis was conducted using STRING (http://string-db.org) [43], which is an online database for predicting functional interactions between proteins. In this study, we uploaded DEGs to the STRING database, then selected with the threshold of combined score > 0.4 to perform the PPI network analysis. Then, the Cytoscape software (http://www.cytoscape.org/, version 3.7.2) [44] was used to visualize and construct the transcriptional regulatory network of common DEGs. Each node in the diagram of Cytoscape represents a gene or protein, and the connections between nodes constitute the interaction relationship between the molecules. Usually, nodes with the greatest numbers of interactions with neighboring nodes were considered hub nodes. We used the Cytohubba plugin to sort genes according to the maximum correlation criterion (MCC), and the top 20 genes in the score results were determined as the hub genes. Generally, hub genes are deemed to play a critical role in the function and have high correlation with other genes.

### Regulatory network analysis

In order to construct the gene-miRNA regulatory network, the online prediction tools miRWalk (http://zmf.umm.uni-heidelberg.de/apps/zmf/mirwalk/) [45] was applied to predict the possible target miRNAs. MiRWalk is a comprehensive archive, supplying the largest available collection of predicted and experimentally verified microRNA (miRNA)-target interactions with novel and unique features. The intersection of miRNA predicted by miRWalk and miRTarbase were chosen as the analysis prediction result, and the screening conditions were required in the following criteria: minimum seed sequence length is set to 7 mer, *P* < 0.05, and the target gene binding region was 3′UTR. The gene-miRNA pairs that have a reverse transcription expression relationship, were incorporated into the interactive network construction and visualized with Cytoscape software.

### Single cell RNA data analysis

The Gene Expression Omnibus (GEO) database was used to obtain the expression matrix data for single-cell transcriptome profiling. We used the Seurat package to create the object and filtered out low-quality cells. After that, we did standard data preprocessing, calculating the percentage of gene numbers, cell counts, and mitochon-dria sequencing count. We eliminated genes with fewer than three detected cells and genes with fewer than 300 detected gene numbers. After data filtering, these steps were made: Identification of highly variable features (feature selection); Scaling the data; Perform linear dimensional reduction; Determine the ‘dimensionality’ of the da-taset; Cluster the cells; Run non-linear dimensional reduction (UMAP); Finding differ-entially expressed features (cluster biomarkers); Assigning cell type identity to clusters. Finally, we use a violin plot to show the expression of different groups of hub genes.

### Quantitative real-time PCR

Peripheral blood was collected from 5 patients before and after AIT. Total RNAs of cells were extracted by Trizol reagent (Invitrogen) and converted into complementary DNA (cDNA) using the PrimeScriptTMRT Master Mix (Takara, China) according to the manufacture’s protocol. Then, TBGreen® Premix Ex TaqTM II (Takara, China) was used to perform qRT-PCR. The reaction system (20 µL) was as follows: cDNA 1.6 µL, 2 × TB Green 10 µL, RNase-free water 6.8 µL, upstream and downstream primers 0.8 µL each. Each sample was replicated three times. The experimental procedure was as follows: 95 °C for 3 min, 95 °C for 5 s, 60 °C for 30 s, 95 °C for 10 s, totaling 40 cycles. The relative expression was computed using the 2^−ΔΔ^CT method. Prism8.0 was used to plot significant differences after analyzing them with SPSS23.0 software.

### Statistical analysis

To compare continuous variable differences between groups, the independent-sample t-test, ANOVA, Mann-Whitney test, Wilcoxon signed ranks test, and Kruskal-Wallis test were used. To calculate the correlation coefficient between ranked variables, the Spearman rank-correlation test was used.

## Data Availability

The datasets generated for this study can be found in the Gene Expression Omnibus database (GEO, www.ncbi.nlm.nih.gov/geo/):GSE37157, GSE29521 and GSE200107.

## Abbreviations

AR: Allergic rhinitis
AIT: Allergen-specific immunotherapy
SCIT: Subcutaneous immunotherapy
SLIT: Sublingual immunotherapy
DEGs: Differentially expressed genes
PBMC: Peripheral blood mononuclear cell
UMAP: Uniform manifold approximation and projection
